# Metastatic Breast Cancer Masked as Constipation

**DOI:** 10.7759/cureus.16031

**Published:** 2021-06-29

**Authors:** Edwin McCray, Robyn Naron, Sarah White, Sarah Messersmith, Christopher Stewart

**Affiliations:** 1 Internal Medicine, Campbell University School of Osteopathic Medicine, Lillington, USA; 2 Internal Medicine, Harnett Health System, Lillington, USA

**Keywords:** metaplastic breast cancer, breast cancer screening, paraneoplastic syndromes, hypercalcemia, early detection of cancer

## Abstract

Even though screening mammography has been attributed to decreased mortality in recent decades, breast cancer is one of the leading causes of death among women in the United States. Disruption of screening protocols and variation in the presentation may alter the course of detection and management. We report a case of hormone receptor-positive breast cancer that presented as vague gastrointestinal symptoms in a patient with a delayed workup for a self-discovered breast lump during the coronavirus disease global pandemic.

A 48-year-old woman with a history of gastroesophageal reflux and hypertension presented to the emergency department with primary complaints of constipation and abdominal distention with associated flatus and nausea. Vitals were within normal limits, and physical examination was notable for abdominal distention and diffuse tenderness to palpation. Labs demonstrated hypercalcemia and an unremarkable complete blood count. A chest X-ray showed a right hilar mass, and a CT chest revealed multiple lytic bone lesions diffusely scattered throughout the entire skeleton; no hilar mass was noted on the CT chest. A CT scan of the abdomen and pelvis incidentally revealed a right breast mass. A bone marrow biopsy identified invasive ductal carcinoma. Mammography and biopsy of the breast mass identified estrogen receptor/progesterone receptor-positive invasive ductal carcinoma, consistent with the bone marrow biopsy, confirming the diagnosis of metastatic breast cancer.

Unpredicted disruptions in screening processes may result in delayed cancer diagnoses. This case illustrates the importance of routine self-breast examinations, screening mammography, and maintaining a broad differential diagnosis.

## Introduction

Breast cancer is the second leading cause of cancer-related deaths among women in the United States [1]. Although mortality has decreased over the past 30 years, the contribution of screening versus improved treatment towards reducing mortality remains uncertain [1]. Mammography is the primary screening modality for the detection of breast cancer [2]. Patients are encouraged to be aware of their own breast anatomy which may lead to earlier recognition of abnormalities.

A palpable breast lump is the most common symptom among women presenting with breast cancer and is relatively highly correlated with the odds of malignancy [3,4]. However, it is important to understand that advanced malignancies can present as paraneoplastic syndromes, such as hypercalcemia, even when breast anatomy has not noticeably changed [5]. We describe the case of a middle-aged woman with seemingly benign gastrointestinal complaints that were ultimately found to be the presentation of hormone receptor-positive breast cancer.

## Case presentation

**Figure 1:**
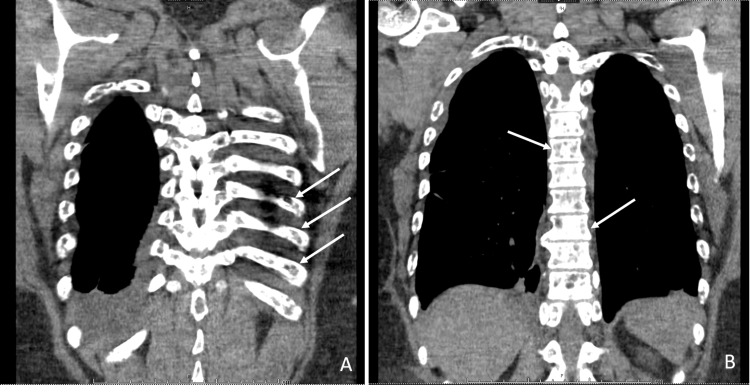
CT chest. CT chest showing diffuse lytic bone lesions in the ribs (A) and spinal column (B), with no evidence of a right hilar mass. CT: computed tomography

**Figure 2:**
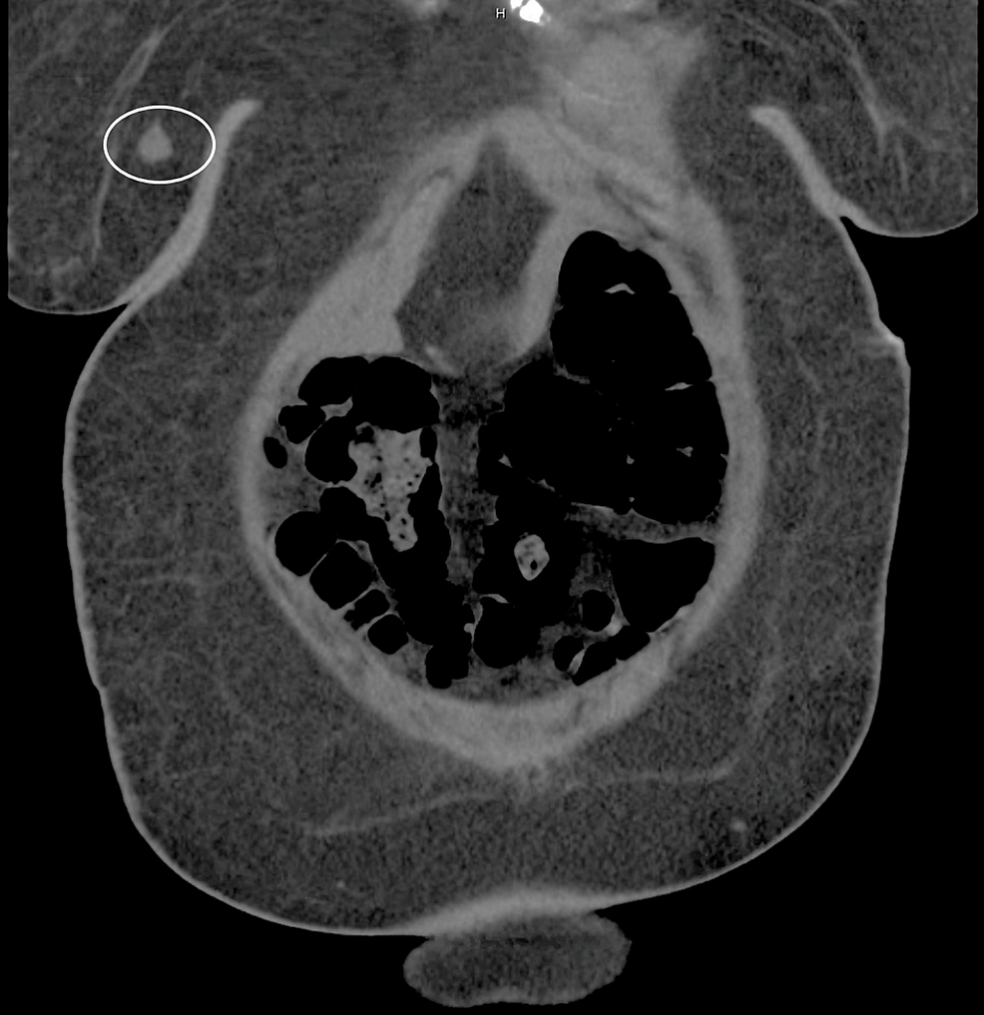
CT abdomen and pelvis. CT scan of the abdomen and pelvis demonstrating evidence of a right breast mass (circled). CT: computed tomography

Ms. A is a 48-year-old G4P4A0 woman with a medical history of gastroesophageal reflux and hypertension who presented to the emergency department with complaints of constipation and abdominal distention for two weeks. Her primary symptoms were associated with flatus, nausea, and abdominal swelling to the point that she questioned if she was pregnant; however, no pregnancy test was ordered in the emergency department. Made evident by observation through future episodes of care, she likely was not pregnant at the time of presentation or anytime since. The patient noted that she had been taking an oral medication for reflux and bloating but could not remember the name of the medication. A review of her systems was otherwise unremarkable, as was her family medical history. Her vital signs were within normal limits. Physical examination was notable for abdominal distention and diffuse tenderness to palpation. A complete blood count with differential was only significant for mild normocytic anemia, and a comprehensive metabolic panel was significant for hyponatremia (129 mEq/L), hypokalemia (3.1 mEq/L), hypochloremia (94 mEq/L), elevated blood urea nitrogen/creatinine ratio (25/1.85 mg/dL), and hypercalcemia (12.8 mg/dL). Initially, the hyponatremia quickly corrected with the administration of intravenous fluids. The abdominal pain was treated with oral milk of magnesia, and an X-ray of the kidneys, ureters, and bladder was ordered, which confirmed constipation. Her constipation and acute kidney injury quickly resolved with intravenous fluids and milk of magnesia, respectively. Initially, her findings were thought to be attributed to milk-alkali syndrome; however, the hypercalcemia persisted and was found to be non-parathyroid hormone (PTH) mediated. A chest X-ray showed a right hilar mass, and a CT chest was ordered, which revealed multiple lytic bone lesions diffusely scattered throughout the entire skeleton; no hilar mass was noted on the CT scan (Figures 1A, 1B). The hematology/oncology service was consulted to assist with the workup aimed at determining if the skeletal lesions were due to breast cancer versus multiple myeloma. Metastatic lung cancer was low on the differential due to the absence of risk factors, including smoking, family history, and occupational exposure. A CT scan of the abdomen and pelvis was ordered to assess for other lesions, on which a right breast mass was incidentally identified by the radiologist (Figure 2).

A bone marrow biopsy identified invasive ductal carcinoma which was found to be estrogen and progesterone receptor (ER/PR) positive. Mammography and biopsy of the breast mass identified ER/PR-positive invasive ductal carcinoma, consistent with the bone marrow biopsy, indicating a primary breast cancer with metastatic disease to the skeleton. While discussing the findings with the patient, she disclosed that she had noticed a mass on a self-breast examination several months prior to admission. However, her scheduled mammography was canceled and rescheduled several times due to office closures and limited outpatient testing early on during the coronavirus disease 2019 (COVID-19) pandemic. During a physical examination of the right breast, she was noted to have a 1 cm, hard, irregular, poorly circumscribed nodule in the 5 o’clock position without tenderness, skin dimpling, or palpable lymphadenopathy. The patient was discharged and has been following with the Harnett Health Cancer Center for chemotherapy and hormone treatment with Zoladex and letrozole.

## Discussion

The benefit of breast cancer screening in women aged 40 to 49 years is an area of debate among researchers and clinicians [6]. The World Health Organization advises that screening in women aged 39 to 49 is beneficial, while the Canadian Task Force on Preventive Health Care advocates against this practice, citing that it poses more risk than benefit for asymptomatic women [6]. The current American College of Physicians recommendation for routine mammograms is only for women aged 50 to 74 at average risk [6]. Different from the mammogram recommendations, The American Cancer Society (ACS) and the United States Preventive Services Task Force recommended that physicians begin discussing mammograms with their patients at age 40 [6]. These screening guidelines are highly studied and significantly improve outcomes [6,7]. The ACS suggests that regular self-breast examinations offer no benefit, but breast awareness including the recognition of abnormalities is crucial for early detection of breast cancer [7].

Our patient, age 48, reported a lump of concern on her self-breast examination and requested a mammogram. Despite the lack of unity on early screening practices in those aged 40 to 49, the clinical guidelines for the assessment of women with a palpable mass are less controversial. The American College of Radiology advocates for diagnostic mammograms in any woman aged 40 or older with a solid, palpable mass as these women are at two-fold increased risk of breast cancer [8,9]. Our patient was scheduled for a mammogram, but due to logistic issues resulting from the COVID-19 pandemic, her appointment was rescheduled. A study by Kaufman et al. suggests that weekly breast cancer diagnoses fell by more than 50% in March and April 2020 due to a reduction in screening practices [10]. Despite the unprecedented barriers posed by the COVID-19 pandemic, breast cancer screening is key for early intervention and detection of breast cancer and should not be undervalued [10].

Many professional organizations, such as the American Society of Breast Surgeons, advocated delaying nonurgent screening mammograms during COVID-19 to protect the safety of both patients and staff [11]. Three priority classes were formed for screening guidelines [11]. Patients were categorized by provider discretion as Priority A, B, or C: critical with an immediate need of screening, noncritical with a need for screening, and able to delay screening, respectively [11]. Our patient fell within Priority B including patients who are in definite need of a screening mammogram, but who are nonurgent and may delay screening for the short term, but not indefinitely. However, due to the rescheduling of her mammogram, our patient was diagnosed with breast cancer only after presenting to the emergency department for seemingly unrelated symptoms.

Various cancers can indirectly cause symptoms across other body systems due to substances released by tumor cells or immune responses to those cells, a phenomenon known as paraneoplastic syndromes [12]; these symptoms may even appear before indications directly related to the malignancy [12]. Breast cancer commonly leads to hypercalcemia of malignancy, a paraneoplastic syndrome by which PTH-related protein is secreted by malignant cells and acts to inappropriately signal for the release of calcium from bone [13]. Hypercalcemia, of any origin, may result in various symptoms related to renal, neurological, and gastrointestinal dysfunction [13]. Of the gastrointestinal-related symptoms, constipation is common [13], which was seen in this case. The clinical presentation along with non-PTH-mediated hypercalcemia seen in this case is consistent with hypercalcemia of malignancy. As with other paraneoplastic syndromes, presentations as such may lead to delayed diagnosis or misdiagnosis of the underlying malignancy.

## Conclusions

This case illustrates the importance of routine self-breast examination, screening mammography, and keeping a broad differential diagnosis. Although this patient presented with a seemingly benign complaint of constipation, further workup revealed evidence of humoral hypercalcemia related to malignancy. The self-discovery of a small, nonmobile, nontender mass should be immediately followed with a mammogram. Unpredicted disruptions in screening processes, as seen in this case due to the COVID-19 pandemic when primary care physicians were placing most nonacute in-person visits on hold and temporarily transitioning to telemedicine, may prove catastrophic in terms of delayed cancer diagnoses. It is likely that Ms. A was not the only patient who missed routine cancer screening during this time.
